# Supplementary material for: Post-transcriptional reprogramming by thousands of mRNA untranslated regions in trypanosomes
Source: Nat Commun. 2024 Sep 16;15:8113. doi: 10.1038/s41467-024-52432-0 (PMC11405848; doi:10.1038/s41467-024-52432-0)
Supplement: Supplementary file 1 — Supplementary Information [file 41467_2024_52432_MOESM1_ESM.pdf]

Post-transcriptional reprogramming by thousands of mRNA untranslated regions in trypanosomes

Anna Trenaman, Michele Tinti, Richard J. Wall and David Horn

Supplementary Figures 1-4

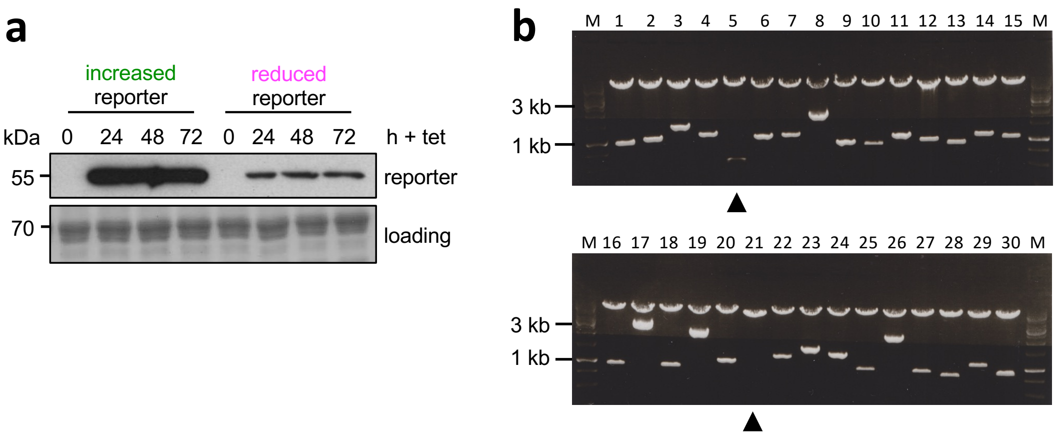

c

$$\frac{\text{average size of library fragments (kbp)} * \text{proportion of clones with inserts} * \text{number of } \textit{T. brucei} \text{ clones generated}}{\text{approx. size of haploid } \textit{T. brucei} \text{ genome (kbp)}}$$

$$\frac{2 * 0.9 * 2{,}500{,}000}{40{,}000} = 112.5 \text{ x coverage} = 56.25 \text{ x forward coverage} + 56.25 \text{ x inverted coverage}$$

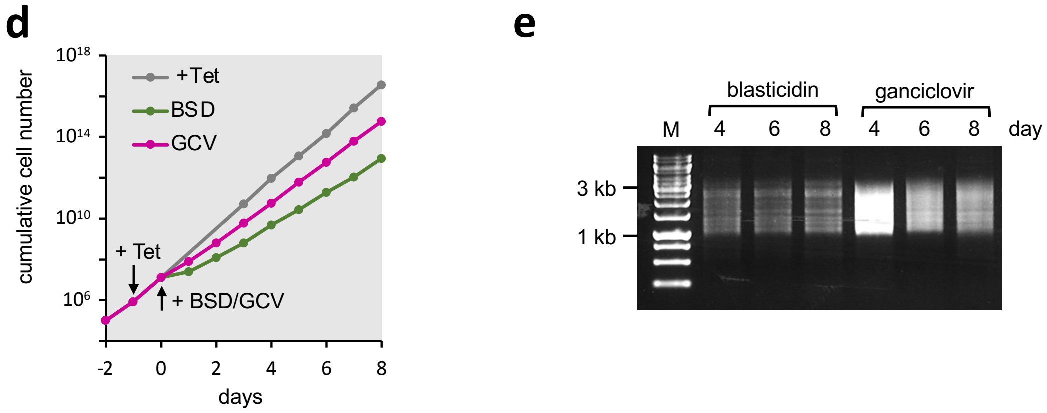

Supplementary Figure 1 | UTR library validation and screening. a Protein blotting demonstrated tetracycline-inducible, stable, and differential expression of the BLA-TK reporter, detected using aTY-1. A Coomassie stained panel provides a loading control. **b** UTR plasmid library subclones were digested with FseI and analysed by agarose gel electrophoresis. Twenty-eight of 30 constructs analysed (93%) contained inserts of 1-3 kbp. Arrowheads indicate constructs with inserts outside this size range. **c** Illustration of how library complexity and genome coverage were calculated. **d** Growth of *T. brucei* library populations were monitored during selection with blasticidin (BSD) or ganciclovir (GCV) or without selection (+Tet). **e** PCR amplicons were generated from *T. brucei* genomic DNA following selection and were analysed by agarose gel electrophoresis, revealing amplicons in the expected 1-3 kbp size-range. These data show aliquots of the samples that were submitted to Illumina sequencing analysis.

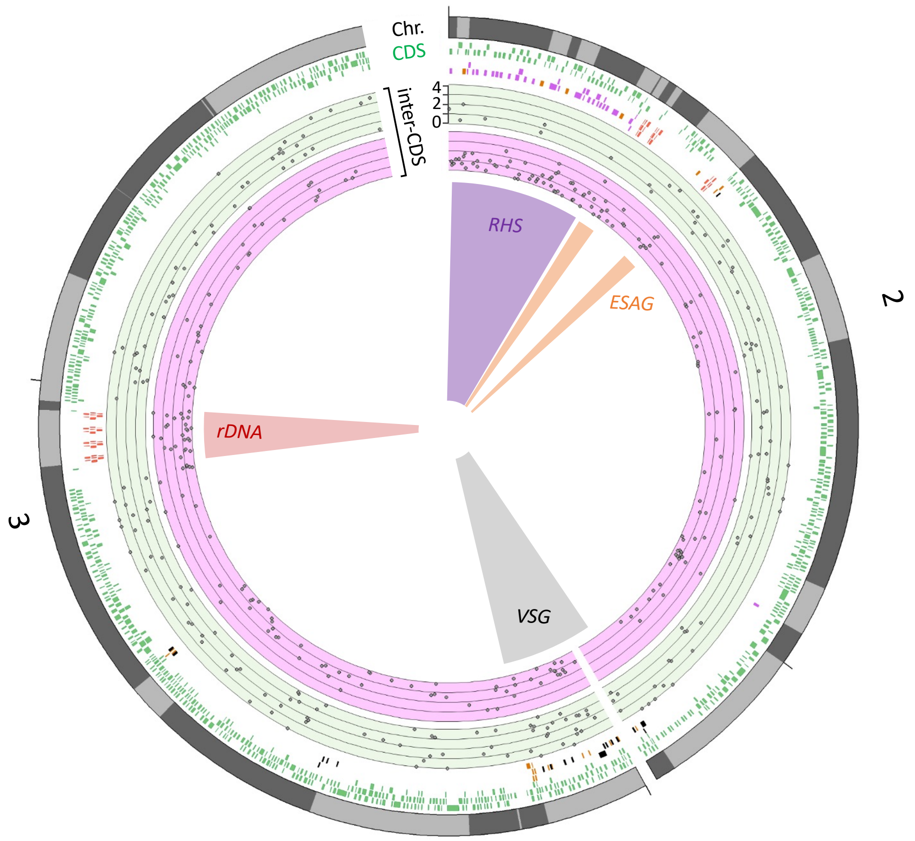

Supplementary Figure 2 | Illustrative UTR-seq hits on chromosomes 2 and 3. The Circos plot shows *T. brucei* chromosomes 2 and 3; approx. 3 Mbp. Polycistrons are indicated on the outer circle. Individual CDSs are shown in green, with variant surface glycoprotein (*VSG*), expression site associated genes (*ESAGs*), retrotransposon hot-spot (*RHS*) and ribosomal RNA (*rRNA*) genes highlighted. Enrichment for inter-CDS DNA fragments inserted in the sense orientation in relation to the reporter that increased (green background) or decreased reporter expression (magenta background) are indicated. Scale is log_2-fold-change relative to control, with values clipped when >4.

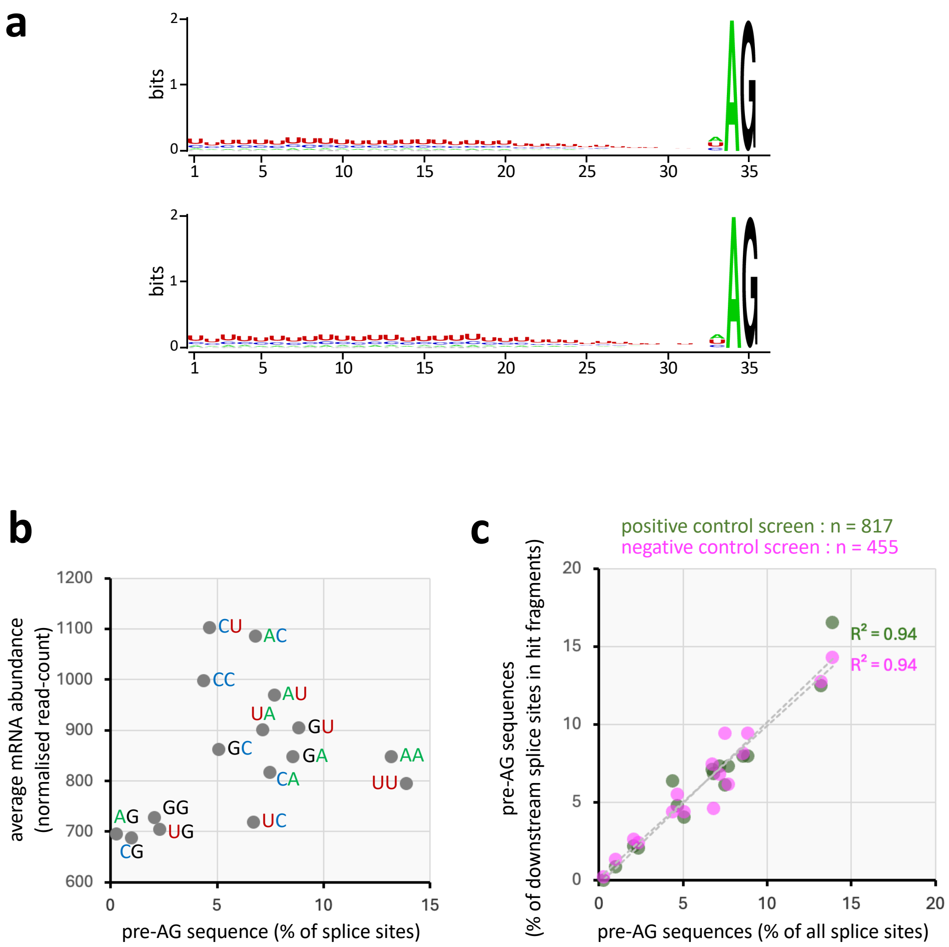

Supplementary Figure 3 | Analysis of *trans*-splicing sites. a The motifs shown were derived from splice-sites associated with the 2000 highest abundance mRNAs (top panel) or the 2000 lowest abundance mRNAs (bottom panel) [3]. **b** The plot shows proportions of splice sites immediately preceded by the dinucleotides indicated, relative to average mRNA abundance. n = 7326. **c** The plot shows proportions of splice sites immediately preceded by the dinucleotides indicated relative to those splice-sites observed downstream of hit fragments in the MPRA.

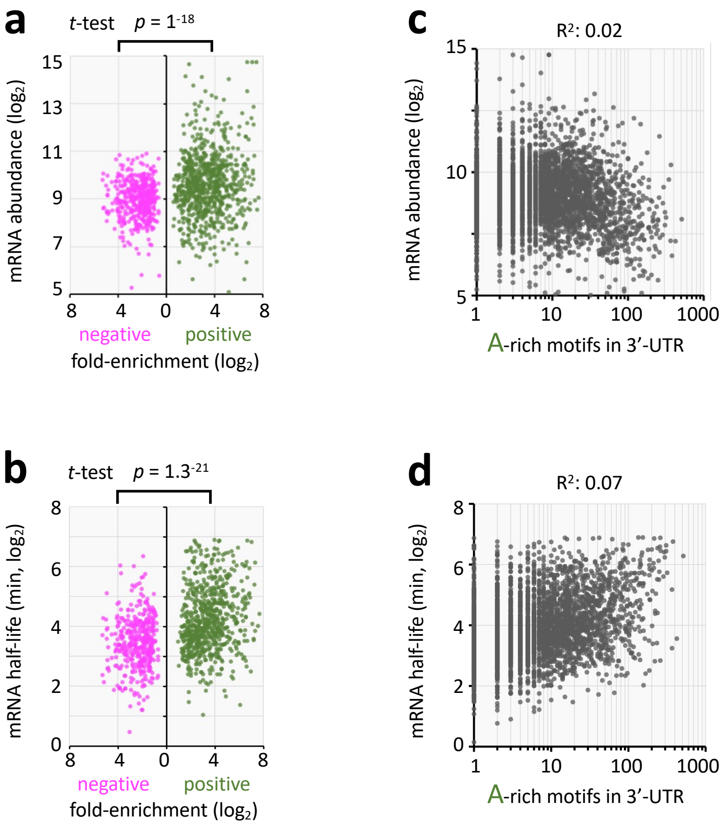

Supplementary Figure 4 | MPRA hit and A-rich motif profiles relative to mRNA abundance and turnover. **a** The plot shows fold-enrichment of hit fragments in the screen relative to published measures of mRNA abundance [3]. **b** As in a but for published measures of mRNA half-life [2]. **c** The plot shows number of A-rich motifs shown in Fig. 4b in 3'-UTRs relative to published measures of mRNA abundance. **d** As in c but for published measures of mRNA half-life. n = 3897. See Figure 4c-d for more details.
