# Supplementary material for: Post-transcriptional reprogramming by thousands of mRNA untranslated regions in trypanosomes
Source: Nat Commun. 2024 Sep 16;15:8113. doi: 10.1038/s41467-024-52432-0 (PMC11405848; doi:10.1038/s41467-024-52432-0)
Supplement: Supplementary file 3 — Description of Additional Supplementary Files [file 41467_2024_52432_MOESM3_ESM.pdf]

Description of Additional Supplementary Information

Supplementary Data 1 | Sheet 1 details the UTR-seq data for the blasticidin, positive control arm of the MPRA for fragments in the native orientation relative to transcription. Coordinates for 3'-UTRs, MPRA fragments, indexed reads and statistical analysis are shown. Sheet 2 details the equivalent data for fragments in the inverted orientation relative to transcription. Sheet 3 details the equivalent data for for the ganciclovir negative arm of the MPRA for fragments in the native orientation relative to transcription. Sheet 4 details the equivalent data for fragments in the inverted orientation relative to transcription. Sheet 5 details the known regulatory T. brucei 3'-UTRs. Sheet 6 details oligonucleotide sequences.
