# Supplementary material for: Post-transcriptional reprogramming by thousands of mRNA untranslated regions in trypanosomes
Source: Nat Commun. 2024 Sep 16;15:8113. doi: 10.1038/s41467-024-52432-0 (PMC11405848; doi:10.1038/s41467-024-52432-0)
Supplement: Supplementary file 5 — Reporting Summary [file 41467_2024_52432_MOESM5_ESM.pdf]

nature portfolio

Corresponding author(s): David Horn

Last updated by author(s): 23 August 2024

Reporting Summary

Nature Portfolio wishes to improve the reproducibility of the work that we publish. This form provides structure for consistency and transparency in reporting. For further information on Nature Portfolio policies, see our Editorial Policies and the Editorial Policy Checklist.

Statistics

For all statistical analyses, confirm that the following items are present in the figure legend, table legend, main text, or Methods section.

n/a	Confirmed	
☐	☒	The exact sample size (*n*) for each experimental group/condition, given as a discrete number and unit of measurement
☐	☒	A statement on whether measurements were taken from distinct samples or whether the same sample was measured repeatedly
☐	☒	The statistical test(s) used AND whether they are one- or two-sided<br>*Only common tests should be described solely by name; describe more complex techniques in the Methods section.*
☐	☒	A description of all covariates tested
☐	☒	A description of any assumptions or corrections, such as tests of normality and adjustment for multiple comparisons
☐	☒	A full description of the statistical parameters including central tendency (e.g. means) or other basic estimates (e.g. regression coefficient) AND variation (e.g. standard deviation) or associated estimates of uncertainty (e.g. confidence intervals)
☐	☒	For null hypothesis testing, the test statistic (e.g. *F*, *t*, *r*) with confidence intervals, effect sizes, degrees of freedom and *P* value noted<br>*Give P values as exact values whenever suitable.*
☒	☐	For Bayesian analysis, information on the choice of priors and Markov chain Monte Carlo settings
☒	☐	For hierarchical and complex designs, identification of the appropriate level for tests and full reporting of outcomes
☐	☒	Estimates of effect sizes (e.g. Cohen's *d*, Pearson's *r*), indicating how they were calculated

Our web collection on statistics for biologists contains articles on many of the points above.

Software and code

Policy information about availability of computer code

Data collection	no software was used
Data analysis	samtools=1.9, a suite of programs for interacting with high-throughput sequencing data; deeptools=3.5.0 a suite of python tools particularly developed for the efficient analysis of high-throughput sequencing data; subread=1.6.4 comprises a suite of software programs for processing next-gen sequencing read data; bedtools=2.29.0: a toolset for genome arithmetic, scikit-learn=0.22 Simple and efficient tools for Machine Learning in Python; statsmodels=0.10.1 is a Python module that provides classes and functions for the estimation of many different statistical models, as well as for conducting statistical tests, and statistical data exploration; circus=0.69-8 a software package for visualizing data and information in a circular layout; - pygraphviz=1.3 a Python interface to the Graphviz graph layout and visualization package. svist4get=1.2.24 is a simple bioinformatics tool for visualization of genomic signal tracks in user-defined genomic windows. MEME=5.5.5 is a software toolkit for performing motif-based sequence analysis, weblogo=2.8.2 a web based application designed to for the generation of sequence logos. Custom software available at https://github.com/mtinti/utr_bsf_code and https://zenodo.org/doi/10.5281/zenodo.10636308.

For manuscripts utilizing custom algorithms or software that are central to the research but not yet described in published literature, software must be made available to editors and reviewers. We strongly encourage code deposition in a community repository (e.g. GitHub). See the Nature Portfolio guidelines for submitting code & software for further information.

Data

Policy information about availability of data

All manuscripts must include a data availability statement. This statement should provide the following information, where applicable:

- Accession codes, unique identifiers, or web links for publicly available datasets
- A description of any restrictions on data availability
- For clinical datasets or third party data, please ensure that the statement adheres to our policy

High-throughput sequencing data generated for this study have been deposited at the Sequence Read Archive under primary accession number PRJNA1076082 (https://www.ncbi.nlm.nih.gov/sra/PRJNA1076082). Source data are provided with this paper in the Source Data file and in Supplementary Data file 1.

Research involving human participants, their data, or biological material

Policy information about studies with human participants or human data. See also policy information about sex, gender (identity/presentation), and sexual orientation and race, ethnicity and racism.

Reporting on sex and gender	N/A
Reporting on race, ethnicity, or other socially relevant groupings	N/A
Population characteristics	N/A
Recruitment	N/A
Ethics oversight	N/A

Note that full information on the approval of the study protocol must also be provided in the manuscript.

Field-specific reporting

Please select the one below that is the best fit for your research. If you are not sure, read the appropriate sections before making your selection.

☒ Life sciences ☐ Behavioural & social sciences ☐ Ecological, evolutionary & environmental sciences

For a reference copy of the document with all sections, see nature.com/documents/nr-reporting-summary-flat.pdf

Life sciences study design

All studies must disclose on these points even when the disclosure is negative.

Sample size	Sample size was chosen based on the use of positive or negative selection, with samples taken on days 4, 6 and 8. Given a typical cell-doubling time of 6-7 h, this was considered sufficient to generate a dynamic range of downstream read-counts to identify positive or negative regulatory DNA fragments in each assay.
Data exclusions	There were no data exclusions.
Replication	Read-counts were compared pairwise between day-4, day-6 and day-8 blasticidin and ganciclovir selected samples. Indexed reads from opposite ends of each fragment were considered separately, yielding six pairwise comparisons. These values served as the basis for the two-sided Wilcoxon rank-sum test, for computing log2 fold changes and log2 average intensity. p-values obtained from the Wilcoxon test were adjusted for multiple comparisons using the False Discovery Rate Benjamini-Hochberg method.
Randomization	Randomisation was not considered necessary.
Blinding	Blinding was not considered necessary for a massive parallel assay, where the behavior of individual clones was dependent upon common, 'internally controlled' growth conditions.

Reporting for specific materials, systems and methods

We require information from authors about some types of materials, experimental systems and methods used in many studies. Here, indicate whether each material, system or method listed is relevant to your study. If you are not sure if a list item applies to your research, read the appropriate section before selecting a response.

Materials & experimental systems

n/a	Involved in the study
☐	☒ Antibodies
☐	☒ Eukaryotic cell lines
☒	☐ Palaeontology and archaeology
☒	☐ Animals and other organisms
☒	☐ Clinical data
☒	☐ Dual use research of concern
☒	☐ Plants

Methods

n/a	Involved in the study
☒	☐ ChIP-seq
☒	☐ Flow cytometry
☒	☐ MRI-based neuroimaging

Antibodies

Antibodies used	Mouse monoclonal Anti-Ty1 SIGMA/Merck SAB4800032 clone BB2 1:5,000<br>Mouse HRP-coupled secondary antisera Bio-Rad 1:10,000
Validation	Anti-Ty1 antibody is a monoclonal antibody against the Ty1 epitope tag. Validation against Ty1 tagged protein in Trypanosoma brucei was carried out by running in parallel to induced Ty1 tagged protein expressing cell lysates, an un-induced protein sample that does not express a Ty1 tagged protein. No non-specific signals were observed in this control sample lane.

Eukaryotic cell lines

Policy information about cell lines and Sex and Gender in Research

Cell line source(s)	Trypanosoma brucei brucei Lister 427 was originally obtained from Prof. George Cross (Rockefeller University, NYC, USA). Subsequent genetic modifications were performed by the authors.
Authentication	Not part of the current study, but proteomics and genome sequencing provide authentication. Also microscopy.
Mycoplasma contamination	Mycoplasma contamination check carried our approx. every 5 years - no positive results from those tests to date.
Commonly misidentified lines<br>(See ICLAC register)	T. b. brucei L427 is not a commonly misidentified line.

Plants

Seed stocks	N/A
Novel plant genotypes	N/A
Authentication	N/A
